# Cooperative longevity of interaction model for addressing paused handoff problem in smart city intelligent transportation systems

**DOI:** 10.1371/journal.pone.0318997

**Published:** 2025-03-17

**Authors:** Abdullah Faiz Al Asmari, Tariq Alqubaysi, Fayez Alanazi, Ahmed Almutairi, Ammar Armghan

**Affiliations:** 1 Civil Engineering Department, College of Engineering, King Khalid University, Abha, Saudi Arabia; 2 Department of Civil Engineering, College of Engineering, Northern Border University, Arar, Saudi Arabia; 3 Civil Engineering Department, College of Engineering, Jouf University, Sakaka, Saudi Arabia; 4 Department of Civil and Environmental Engineering, College of Engineering, Majmaah University, Majmaah, Saudi Arabia; 5 Department of Electrical Engineering, College of Engineering, Jouf University, Sakaka, Saudi Arabia; University of Lagos Faculty of Engineering, NIGERIA

## Abstract

Smart cities use Intelligent Transportation Systems (ITS) to manage traffic by continuously communicating with roadside infrastructure and nearby vehicles. Paused handoff interrupts grounded congestion, signal supervision, and path-shifting knowledge. Paused handoffs occur when cars wait to interact owing to volatile neighbours or heavily crowded roadside units. In congested metropolitan areas, ITS vehicle communication interruptions are a significant issue. This research addresses this issue. Hence, the research introduces the Cooperative Longevity of Interaction Model (CLoIM) to enhance communication reliability by minimizing the impact of paused handoff. The model employs a hybrid trained herd optimization algorithm to improve the longevity for interaction between vehicles and roadside units, minimizing handoff interruptions. The approach dynamically adjusts search strategies to prioritize high longevity interactions, improving communication stability. Results show that CLoIM increases longevity by 10.81% and reduces the paused handoff lag by 9.17%, effectively addressing the challenges of vehicle density and mobility in ITS scenarios.

## 1. Introduction

Intelligent Transportation Systems (ITS) regulate traffic by ensuring constant contact between vehicles and roadside infrastructure [1]. Communication handoff ensures seamless engagement, but interrupted handoffs occur when re-establishing connections is delayed owing to traffic congestion [2]. Paused handoffs disturb signal control and vehicle routing, which leads to traffic inefficiencies. Minimizing handoffs is critical for maintaining continuous communication and improving real-time traffic management [3]. Sharing road conditions and signal changes requires good communication. Avoiding disruptions from poor connections or packed roadside units is difficult [4]. Traffic flow is optimized when vehicles are always connected to the network by a suitable handoff mechanism. Preventing these interruptions improves innovative city traffic management systems. The handoff system keeps cars connected across route segments [5,6]. Improving ITS communication lifespan ensures vehicle-roadside data sharing [7]. This reduces communication breakdowns and improves vehicle traffic decisions. The system optimizes communication channels in densely populated areas to maintain smooth connections [8]. Real-time traffic updates, signal changes, and hazard alerts are delivered using this technology. Traffic and infrastructure changes can be accommodated [9]. Regular communication lets vehicles change speed and routes in real-time. The method reduces traffic disruption by preventing dropped connections [10]. Vehicles are maintained up to date on road conditions and traffic, which improves safety and efficiency. Long-term communication improves collaboration between vehicle-to-vehicle and vehicle-to-infrastructure systems [11]. As an added measure, these known sets limit dimensions when creating child solutions [12].

Learning-based models improve the lifespan of communication in ITS by constantly adapting tactics. The technology uses previous data to optimize future transmission scheduling and avoid interruptions [13]. Machine learning algorithms foresee disturbances and react to traffic changes, resulting in steady connections. The approach learns from real-time traffic circumstances to ensure that vehicles and infrastructure communicate reliably [14]. Such learning reduces the likelihood of failed connections and guarantees essential traffic updates are not missed. Learning-based models improve communication in densely populated cities where disruptions are common [15]. The system considers a variety of criteria, including vehicle speed, location, and road conditions. It gradually enhances communication effectiveness, resulting in more fluid vehicle interactions [16]. Such constant learning guarantees that the system responds to environmental changes like weather. The strategy helps to improve traffic management and road safety by ensuring communication stability [17,18]. The main contributions of the paper are listed below:

- The briefing will include some works related to vehicle communication improvement with modern methods addressing different problems.
- The proposed CLoIM is briefed with suitable illustrations, herd optimization implications, training improvements, etc.
- Mathematical models and diagrammatic illustrations of the process flows describe the working and improvements of the algorithms used.
- The discussion using simulation setup, hyperparameters, and metric-based comparisons is presented to verify, validate, and conclude the proposed model’s performance.

To solve spectrum limitations in intelligent transport systems (ITS), Peng et al. [19] created a backscatter device for clandestine communication. The method proposes a symbiotic ITS with ambient backscatter communication to overcome spectrum scarcity and environmental concerns. The system measures performance using detection error probability, outage likelihood, and energy efficiency indicators. The method demonstrates the way adding antennas reduces outages. Zhao et al. suggested a rate maximization model for UAV-assisted vehicular communication networks [20]. The research indicates that optimizing ITS has achieved superior performance in reducing potential traffic accidents on complex road networks [21]. By performing a multi-train system, agents in ITS quickly analyze the error convergence and state constraints under the communication topology [22]. UAVs and RIS will boost communication in dynamic contexts. A deep reinforcement learning system changes UAV trajectories and RIS phase shifts in real-time. Communication and reliability improve extensively using the method.

The rest of the paper is followed by section 3, which discusses the latest literature on the proposed title. The section encompasses details on the proposed cooperative longevity of the interaction model. The results and discussion are given in Section 4. The conclusion is drawn in section 5, and future work is shown in section 6.

## 2. Related works

Li et al. [23] used gradient adaptive optimization to model transportation and show network traffic data transmission. The Energy Efficient Multi-hop Routing (EEMR) algorithm for intelligent transportation wireless sensor networks is described. It uses dynamic cluster-head election and deep learning-based optimization. The method has excellent node survival and low energy consumption, especially during network stress. Intelligent transportation systems’ neural network-based route guidance model was proposed by Zhang et al. [24]. The technique optimizes urban traffic flow utilizing Neural Network-based route guidance. Forecasting trip times and optimizing route selection improves real-time traffic management. It enhances traffic flow and reduces vehicle count variations.

A LoRa-integrated model by Khan et al. [25] improved green transport network connections. IoT applications use LoRa technology for long-range communication to reduce interference and data loss. The dynamic transmission parameter adjustment model optimizes end-device allocation using K-means and DBSCAN clustering. The technology improves network performance and device lifespan. Alqubaysi et al. [26] developed an innovative city transport system driving model to eliminate communication interruptions. The method presents an ITS content delivery framework (CDF) to eliminate communication outages. Content delivery is optimized end-to-end based on vehicle features and environmental conditions. The technique reduces latency and outages.

Jahangeer et al. [27] used cat-swarm optimization to model seamless vehicle network handoffs. Hybrid Cat Swarm Optimization-TOPSIS Algorithm optimizes vertical handoffs in urban vehicle ad hoc networks (VANETs). Adequate mobility and network simulators reduce packet loss and ping-pong handoffs. This enhances handoff performance by enhancing packet delivery and network speed. Vo et al. [28] developed a 5G-enabled ITS traffic flow forecast model. Combining rapid variational mode decomposition, whale optimization, and genetic algorithms predicts traffic flow. Traffic data is modeled using LSTM, Bi-LSTM, GRU, and Bi-GRU. The technique improves forecast accuracy and adaptability to increase prediction accuracy.

Tong et al. [29] proposed a model for information guidance and incentive mechanisms in intelligent transportation systems. The system uses a differentiation concept in traffic rewards and punishments to guide automobiles successfully. The approach modifies information based on vehicle interactions to lower overall transportation costs. The technology stabilizes vehicle behaviour under variable road conditions, improving traffic flow management. Guo et al. [30] introduced a peer-to-peer information exchange approach for vehicle networks. The technique employs a two-tier vehicle ad hoc network (VANET)/peer-to-peer architecture to enhance information exchange in intelligent transportation systems (ITS). The method improves lookup processes using link time, relative velocity, and peer location. The technique improves the efficiency of information exchange as well as system stability.

Ji et al. [31] presented a multi-relay cognitive network model for ITS communication. The solution increases ITS signal transmission reliability with an anti-fragile communication strategy. Energy harvesting and cognitive radio (CR) technology systems have fewer outages with the technique. The process improves gearbox reliability. A priority-based technique for warning message distribution in vehicle networks was presented by Abbas et al. [32]. A priority-based direction-aware media access control (MAC) technique improves vehicle ad hoc network clock synchronization. The system uses a three-tier priority assignment. Message loss and network throughput improve to ensure network message delivery.

Wang et al. [33] used convolutional neural networks to optimize ITS logistics. The technology optimizes ITS logistics with CNNs. The method extracts patterns from accelerometer, gyroscope, and magnetometer data. Logistics operations increase with high categorization accuracy. A 6G intelligent transportation system communication model with UAVs was proposed by Liu et al. [34]. UAV-enabled intelligent offloading reduces energy consumption in ITS IoT devices. Task-gathering places are carefully selected to limit UAV flying distance and energy use. It boosts energy efficiency and performance.

Raza et al. [35] proposed an unmanned aerial vehicle (UAV)-assisted communication model for urban traffic networks. The technology combines micro-UAVs with vehicle ad hoc networks (VANETs) to improve communication in urban settings with low signal strength. The approach uses UAVs’ line-of-sight communication capabilities to ensure reliable connections. The approach increases throughput and eliminates delays in safety and entertainment applications. Ali et al. [36] designed a software-defined network (SDN)-based emergency message routing architecture. The system combines digital twin technology with SDN to route emergency messages in vehicle ad hoc networks (VANETs). The method includes a dependable emergency message routing schema that predicts vehicle positions to improve routing selections. The strategy increases network performance and packet delivery rates.

Kim et al. [37] presented an optimization-based smart camera deployment strategy for urban traffic monitoring to improve efficiency under fluctuating traffic loads. Two approaches optimize single-lens camera placement for fluid surveillance. The collection covers traffic volume and the positioning of cameras scenarios simulated extensively. Results show increased monitoring coverage and traffic flexibility. The model’s assumptions of perfect environmental circumstances may not account for real-world variables like weather or traffic delays, reducing its practicality.

Lee et al. [38] presented a secure framework for IoT-assisted Maritime Transportation Systems (MTS) using virtual emotion detection and differential security barriers. Using mobile robots and UAVs to maximize security barriers improves priority area security. Two approaches use changing security obstacles to optimize security awareness across MTS subareas. Simulations show that the proposed strategies improve maritime security in many circumstances.

## 3. The proposed cooperative longevity of interaction model

The detection of paused handoff issues in smart cities is addressed to ensure reliable data for transportation. This approach relies on vehicle-to-vehicle communication, which experiences congestion and time lag. These issues are addressed by proposing a hybrid trained herd optimization algorithm commonly used to find fitness conditions such as longevity and higher communication rates. This is retained by introducing a herd optimization algorithm to ensure higher longevity and communication. From this state of the art, the paused handoff is addressed where the training is given accordingly to the preceding vehicle transportation. Vehicle transportation analyses the path changes due to the interruption and congestion. These are reflected from the time lag and observe the path changing for the pathway where longevity is maintained. In this execution step, cooperative communication and interaction lead to higher vehicle longevity. For this computation step, the herd optimization is designed, and it is said to be a group of agents that communicate randomly and are processed in a single direction. The training is given to herd agents if any path change is detected to retain longevity and communication. This step indicates convergence rate detection, where it explores the routing for the traffic road by communicating with the neighbouring vehicle. By examining this process, the initial step is to detect the paused handoff problem and how it is addressed, which is computed in the following equation.


$$\partial =\frac{1}{h_{n}}+\sum_{A_{c}}^{h_{0}}z^{\prime }+\left\{\left[\left(Ve^{\prime }+\pi \right)\right]*\sum_{E_{d}}\left(T^{\prime }+h_{0}\right)\right\}*\left[\left(PH_{0}+L_{g}\right)+\left(c_{x}+h_{0}\right)*T^{\prime }\right]+\sum \left(Ve^{\prime }+T'\right)*r_{n}-G_{m}$$ (1)

**Fig 1:**
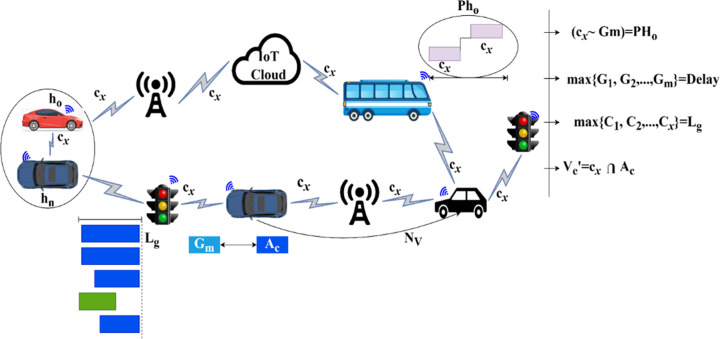
Implication and illustration of the variables in ITS.

From the above equation, the examination is done for vehicle-to-vehicle transportation. The approach indicate she detection of the vehicle and ensures the optimization; the examination is ∂, $h_{0}$ are the vehicle and *n* number of vehicles is $h_{n}$. The cooperative data is labelled as $A_{c}$, the optimization is z', and the paused handoff is represented as $PH_{0}$. Longevity is ensured in this work, and it is described as $L_{g}$. The communication is $c_{x}$, π is the detection of vehicles on roadside units. Based on this, ITS is used to communicate with the neighbouring vehicle, and ITS is T'. The preceding is $E_{d}$, $r_{n}$is denoted ads retained communication and longevity, whereas $G_{m}$ is symbolized as lag time, and the convergence is Ve'. Based on this process, the paused handoff is detected, reducing the vehicle communication and interaction. The implication of the above equation in the ITS scenario with variable illustration is presented in Fig 1.

This illustration in Fig 1 is used to understand traffic detection on the side and longevity and communication computations. Based on this section of examination of paused handoff, the signal control and path changing are interrupted. It leads to miscommunication and interaction failure, increasing the time lag among vehicles. In this state, the lag time indicates the optimization of the vehicle and ensures communication between neighboring vehicles. This case is used to provide reliable communication and interruption and is addressed with the paused handoff condition. This step is processed to detect the traffic in smart cities and observe the longevity of the vehicles. The evaluation runs to retain the longevity and communication $\left\{\left[\left(Ve^{\prime }+\pi \right)\right]*\sum_{E_{d}}\left(T^{\prime }+h_{0}\right)\right\}$. After examining the paused handoff problem, the analysis for cooperative longevity and interaction with vehicles is done and is equated below.


$$\rho =\prod_{h_{n}}^{h_{0}}\left(c_{x}+N_{v}\right)*\left|\left(r_{n}+\left(L_{g}+c_{x}\right)\right)\right|+\left\{\left(PH_{0}+ch_{i}\right)+\left(A_{c}+N_{v}\right)\right\}*o^{\prime }-G_{m}+\prod_{\partial }T^{\prime }+\left(h_{n}*PH_{0}\right)-Ve^{\prime }+\left(ch_{i}*E_{d}\right)$$ (2)

The analysis is done for the cooperative findings of longevity interaction with the vehicle; it is used to define the higher communication analysis as ρ. The communication is stabilized among the vehicles, and the neighbouring vehicles are $N_{v}$, the path changing is represented as $ch_{i}$, which is defined as the traffic on the roadside unit, transportation o'. This observation step includes the convergences and interruptions due to the lack of neighbours/ communication slots. These are sorted out in this proposal and ensure the interaction without any time lag. It indicates the longevity and communication and addresses the paused handoff. If the paused handoff is detected, the preceding data is mapped and processed for the path change. This path change occurs due to traffic, and at this point, herd optimization works to prolong the interaction and transportation.

The transportation of data leads to communication without any convergence or interruption. From this approach, path changing occurs if traffic is detected in smart cities and established securely for communication. Thus, both the $c_{x}$ and $L_{g}$ are maintained and mapped with the preceding data from ITS. In preceding this observation, the paused handoff is detected at the desired checkpoint and reduced. Thus, the scope of work starts with fixing the paused handoff on the roadside unit and evaluating the fitness value. Based on this neighboured vehicle communication takes place without the path changing, and it is formulated as $\left\{\left(PH_{0}+ch_{i}\right)+\left(A_{c}+N_{v}\right)\right\}$. This approach is introduced to transport the data to the neighboured vehicle without time lag. Here forth, the communication and path changing are observed, which leads to interruption, so detection occurs due to paused handoff, which is derived below.


cx=Nv+h0*o′∂+Lg+∥ρ+π−Ve′∥−Gm*o′+h0+T′Lg*∑Edrn+chi*ρ+Ve′−o' (3)

Communication is observed among the data sharing or transportation between the vehicles and ensures longevity. This case deals with the herd optimization that assigns the new agent if path changes occur. Due to this observation step, the convergence rate is reduced if the paused handoff is fixed. These two are directly proportional to each other, where transportation is processed in terms of communication and interaction. Both communication and interaction are addressed to benchmark the longevity of vehicles in smart cities. This plays a role in detecting communication between the vehicles by decreasing the time lag. It states the preceding data processing under ITS and provides information on whether the path change should occur. Based on the result, the communication is followed up, and both longevity and communication are retained. This step of observation relies on the finding of interruption.

Based on this detection phase, communication is established, and interruptions are decreased. The longevity is maintained to reduce the convergences and paused handoff. The handoff is addressed, optimization is developed for this, herd, and communication is followed up. The path changing is done if the interruption or traffic is observed on the road $\pi +\left(h_{0}*c_{x}\right)$. This case is used to envelope data transportation among the vehicles and relies on paused handoff detection. If the paused handoff is detected, it results in interruption and convergence. Based on these two parameters, the detection phase is carried out for the convergences and interruption $\pi +\left(h_{0}*E_{d}\right)*\sum_{Ve'}\partial$. Thus, the detection is carried out for the communication and path-changing factor, and from this, the initiating time and longevity of the interaction are computed using a hybrid trained herd optimization algorithm to ensure paused handoffs are less or null.

### 3.1. Hybrid trained herd optimization

**Fig 2:**
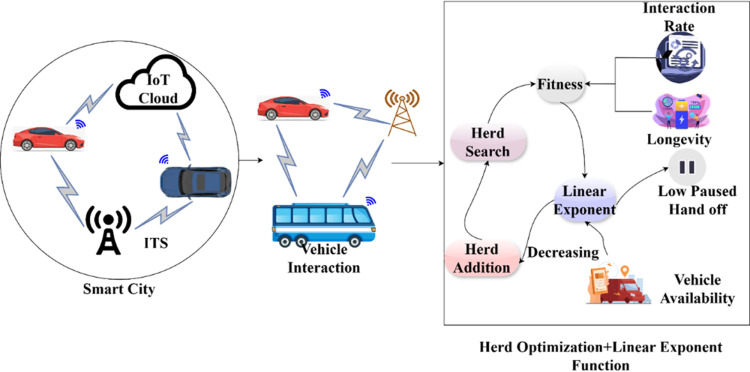
Overall process illustration of herd optimization.

The integration is processed among the herd agents to optimize vehicle communication and interaction and avoid congestion where longevity and communication increase for the pathway changes. This optimization discusses transportation parameters and adaptability to observe the paused handoff and evaluate whether it is less or null. The overall herd optimization process is presented in Fig 2.

The herd optimization identifies $N_{v}$ from the $c_{x}$ region with $Ch_{i}$ and Ve' estimation. The fitness for each herd agent is computed using ρ [equation (2)] provided $L_{g}$ is high. The linear exponent process validates this equation-based output to ensure maximum vehicle availability. This exponent variation decides the augmentation of new herd agent/ deciding the low $PH_{o}$. Therefore, the influencing parameters are $(A_{x}$ and $r_{n}$) for which $G_{m}$ is less and Ve' is high. Thus, herd optimization relies on $\left(h_{n},\;c_{x}time,\;T^{\prime },\;andG_{m}\right)$ inputs for maximizing Ve' (Fig 2). The below equation is used for the training section of the herd agent for optimization results and to find whether it is the lesser or null value.


$$g^{\prime }=\pi +\left(T^{\prime }*c_{x}\right)+ch_{i}*\sum_{h_{0}}^{h_{1}}\left(Ve^{\prime }+PH_{0}\right)*\left\{\left[\left(E_{d}+L_{g}\right)+\left(a_{c}+h_{d}\right)\right]*N_{v}\right\}-G_{m}+\left[\left(PH_{0}*A_{c}\right)+h_{n}\right]*r_{n}\left(L_{g}+c_{x}\right)$$ (4a)

The above equation is generated for the training section under herd optimization and ensures the higher longevity of vehicle transportation. The training is g', $h_{d}$ represents the herd agent that is generated if any new pathway changes occur. This is due to the traffic and congestion on roadside units, which degrades transportation at respective time intervals. Based on this processing step, the preceding factor is initialized for routing the vehicle in smart cities and observes the interaction. The training uses the prior value in ITS and provides the vehicle’s current status on the roadside. From this, herd optimization generation and computation are evaluated if pathway changes occur and move in a single direction. Post to this step, the adaptability for transportation is derived in the following section.


$$B_{p}=\left(g_{a}*g'\right)+\left[\left(Ve^{\prime }-G_{m}\right)-E_{d}\right]+h_{n}*PH_{0}$$ (4b)

**Fig 3:**
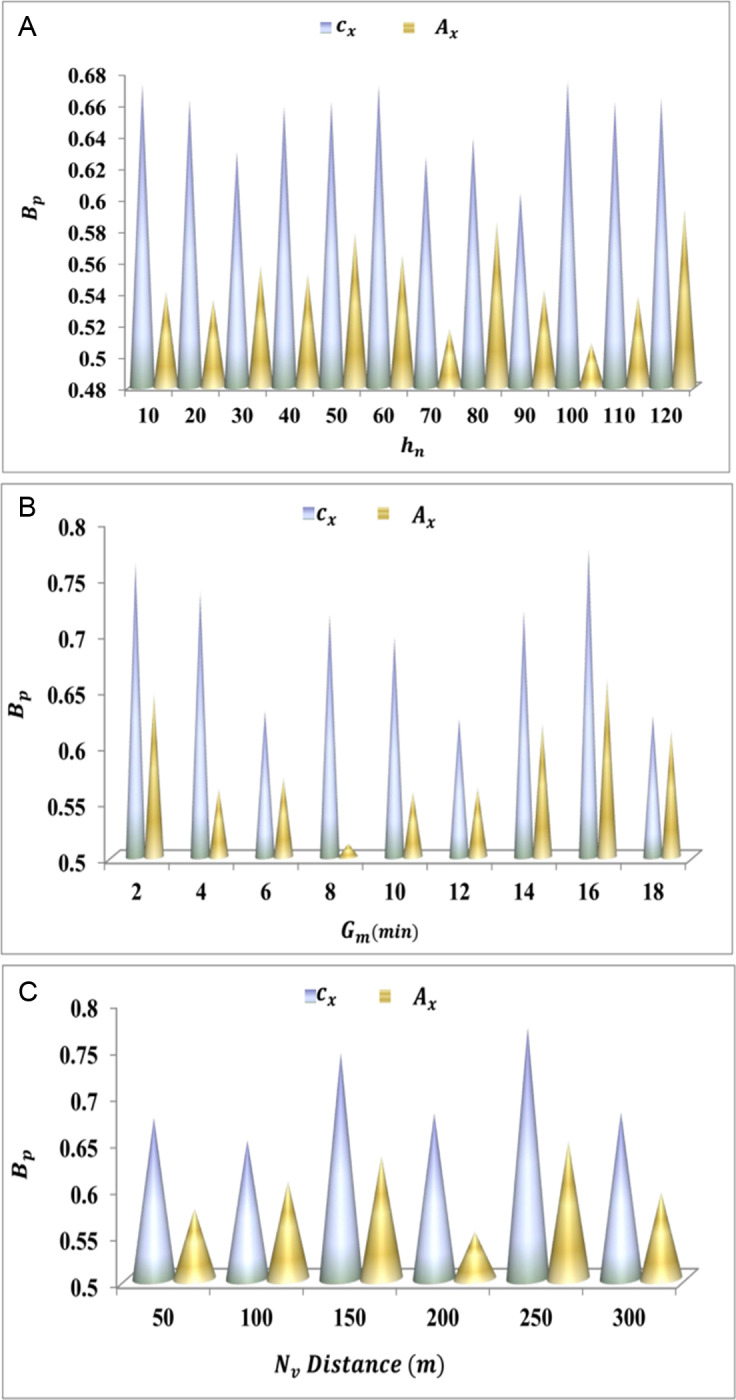
(A–C) $B_{p}$Valuation for $c_{x}$and $A_{x}$.

The adaptability is processed in this derivative, and it is labelled as $B_{p}$, where the matching is carried out and is described as $g_{a}$. This is executed with the preceding value generated on ITS, and mapping is followed up for the cooperative analysis. In this execution step, the vehicle is routed to address convergences and lag time. By executing this step, the paused handoff is done where path-changing is carried out. From this observation step, the path changing is carried out to optimize the resultant by using a herding agent. The herd’s adaptability for $c_{x}$ and $A_{x}$ is valued using Fig 3a–3c.

The adaptability increases if the paused handoff decreases for the addition of a herd agent. This execution step indicates the higher longevity and interaction and identifies the pathway changing based on this herd agent is generated $Y_{i}\left(h_{d}*Ve^{\prime }\right)+A_{c}$. From this examination step, the response time to hand over data is done on the transportation and indicates the higher adaptability among the vehicles $B_{p}+\left(N_{v}*\pi \right)+w_{y}$. In this step, it executes the routing of vehicles if there is traffic is detected $u_{r}\left(T^{\prime }*A_{c}\right)$ Fig 3a–3c. The herd searching is followed up to generate the fitness value, which is equated below.


$$h_{d}\left(I_{s}\right)=\left(B_{p}+N_{v}\right)+\left(a_{c}+c_{x}\right)*\prod_{PH_{0}}L_{g}+\left|Ve^{\prime }-G_{m}\right|-g_{a}-E_{d}\left(t_{i}\right)$$ (5)

**Fig 4:**
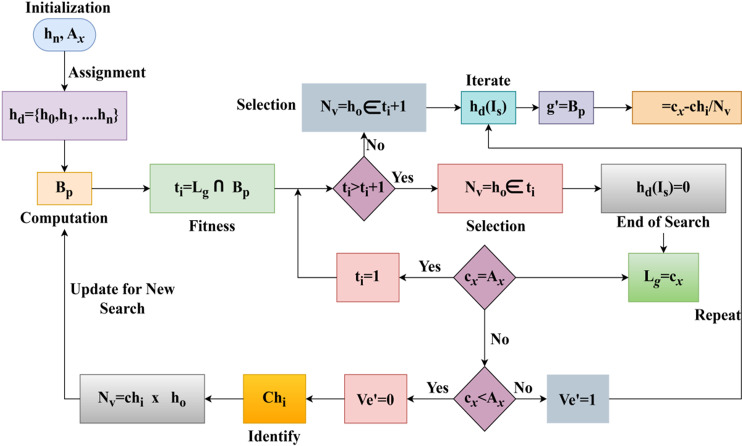
Herd optimization algorithm flow illustrations.

The herd searching is carried out in the above equation, which relies on the training section with the preceding value. The searching is $I_{s}$, where it defines the optimization resultant for the identification of paused handoff. If the paused handoff is detected, retaining The availability is carried forward for the vehicle and ensures higher optimization of herd agent to improve communication and interaction $L_{g}+\left|Ve^{\prime }-G_{m}\right|$. The fitness value is $t_{i}$ generated to increase longevity and communication. The herd pursues a random direction search to identify interacting roadside units or neighbours for communication. This random search is converged using an exponential linear function to improve the selection towards high longevity and, thereby, high interaction rates. The herd optimization algorithm flow is illustrated in Fig 4.

**Fig 5:**
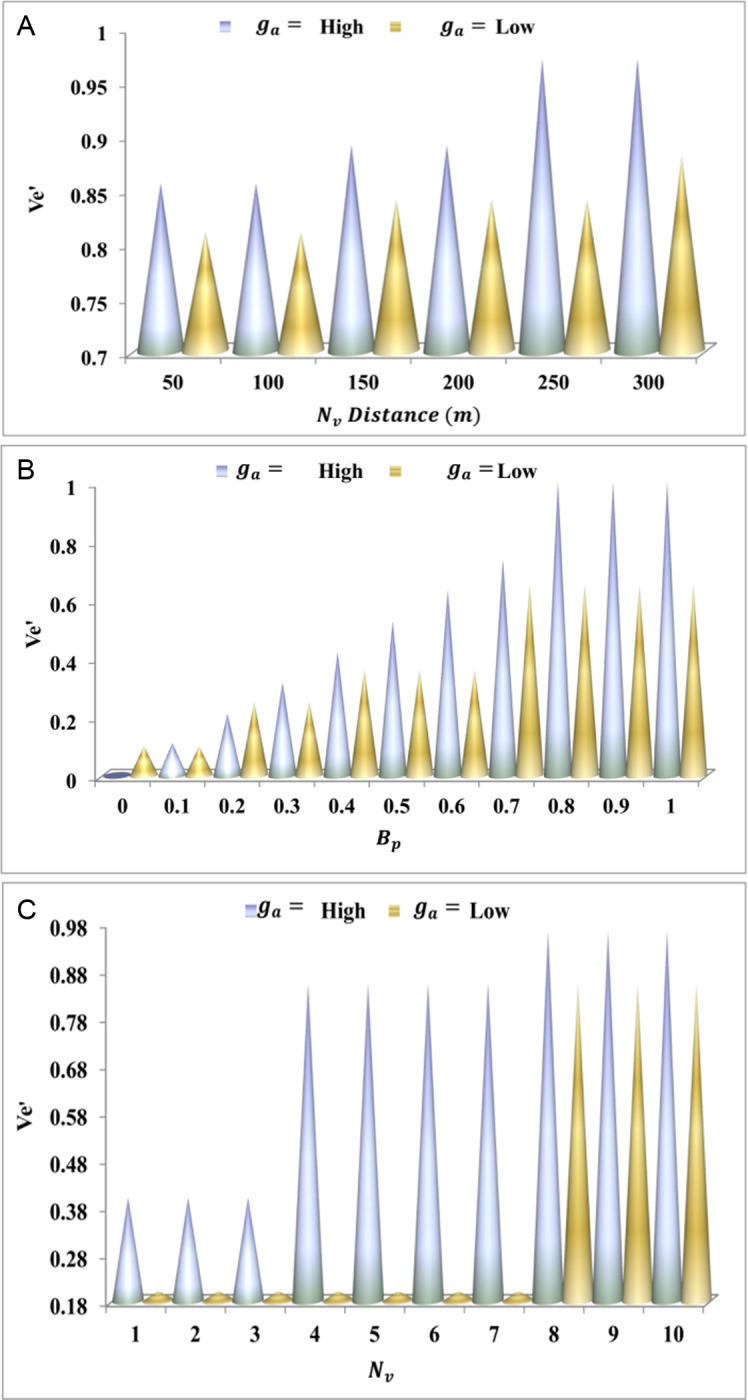
(A–3C) Ve' Valuation for $g_{a}=High$ and $g_{a}=Low$.

The herd optimization flow is depicted in Fig 4 above using the $h_{n}$ and $A_{x}$ inputs. This optimization requires $B_{p}$. During multiple $c_{x}$ intervals where $\left(L_{g}\cap B_{p}\right)$ the joint output-generating process is. The $t_{i}$ and $\left(t_{i}+1\right)$ based $h_{o}\in h_{n}$ are selected to ensure $h_{d}\left(I_{s}\right)$ is not required; this is the case where $PH_{o}=0$. In the $c_{x}=A_{x}$ condition, $t_{i}=1$ for which z' is reached at its maximum; the $r_{n}$ sustained with $B_{p}=$ maximum. The rest of the cases require a $L_{g}$ or Ve' revisit to ensure if $Ch_{i}$ is true or $h_{d}\left(I_{s}\right)$ is required for pursuing communication. These two cases define low convergence and feasibility for any $N_{v}$ that requires an intermediate selection of $h_{o}$ for communication. $V_{e}^{'}=1$ and $\left(N_{v}=Ch_{i}\times h_{o}\right)$ are the iteration and update demanding constraints for $h_{d}$ agents. From this $h_{d}\left(I_{s}\right),$ the Ve' analysis is discussed and presented in Fig 5a–5c.

The convergence increase by retaining longevity and communication and identifying the optimization with the hd agent, which is processed when there are pathway changes. Path-changing occurs where the neighbouring vehicle communicates in this case, whereas the cooperative analysis is done for higher longevity interaction. The optimization is carried out with her agent and improves the convergence rate$Ve^{\prime }+\left(L_{g}+a_{c}\right)*g'$. Thus, the convergences are evaluated for the n number of vehicles $h_{n}*N_{v}+\left(h_{n}+z^{\prime }\right)$ (Fig 5a–5c). This is how the search for the herd is proposed, and this analysis for the linear exponent is followed up in the section below.

### 3.2. Linear exponent description

The linear exponent refers to the result attained from the fitness value where the vehicle availability is detected from the herd searching. This linear exponent is used for the random search and is converged using an exponential linear function to improve the selection towards high longevity and, thereby, high interaction rates. Therefore, the exponential linearity drops the least longevity based on the cooperation time spent by the neighbour or roadside units. By linearly matching the vehicle’s available time based on mobility and longevity, the consecutive herd agents are initiated for new neighbour searches. The preliminary step is to analyze the exponent for mobility and longevity, which is expressed below.


$$\rho =\frac{1}{h_{n}}+\prod_{G_{m}}\left(u_{r}+h_{0}\right)\to N_{v}*w_{y}\left(t_{i}\right)+h_{d}$$ (6)

The analysis is carried out for the mobility check to decrease the time lag when communication is done with the nearby vehicle. The pathway is represented as $w_{y}$, $t_{i}$ is the fitness value. This generation kes place to search for herd optimization and improve mobility, where the consecutive herd agent searches for the new neighbour vehicle. Therefore, mobility is analyzed, and the vehicle availability check is processed using the following equation.


$$h_{0}\left(v^{\prime }\right)=N_{v}\left(I_{s}\right)+h_{d}-w_{y}+o^{\prime }\left(h_{n}\right)-G_{m}-Ve'$$ (7)

The vehicle availability check is followed up to transmit the data to the nearby vehicle where it improves mobility and longevity, and availability is v'. This case illustrates the decreasing of convergences associated with pathway changes. The transportation and new herd search are executed bad on the pathway changes. Based on this, the linear exponent is responsible for transporting the data to the neighboured vehicle without time lag. By processing this, adding a new herd is done if a path change occurs, which is derived below.


$$A_{d}\left(h_{d}\right)=\left(I_{s}-w_{y}\right)\to \rho \left(N_{v}+t_{i}\right)*o^{\prime }\left(h_{d}\right)+z'$$ (8)

The herd addition takes place in the above equation, and it is described as $A_{d}$, where it is used to find the fitness value generated to address the failure of convergences and time lag. Based on this computation step, the availability of the herd agent is initiated, and from that its traffic is detected for the neighbouring vehicle, where mapping is evaluated $g_{a}-E_{d}\left(f_{i}+h_{d}\right)$. This is how the herd addition is done if the linear exponent results in zero value from the fitness and vehicle availability analysis. Post to this process, the linear exponent detection is done for the interaction after herd addition.


$$A_{d}\left(\rho \right)=\sum_{B_{p}}\left(c_{x}+a_{c}\right)*\left[\left(I_{s}+w_{y}\right)*v^{\prime }\right]+h_{n}$$ (9)

The analysis is done to detect herd addition and check whether the interaction is done correctly or not. Based on this step, the failure of convergences is addressed, and the routing is followed up in a training manner. The herd optimization algorithm provides the training, including transportation with neighbouring vehicles. This is meant to periodically check for the interaction after the herd addition and ensure mobility and longevity. Here, identification is done for the interaction on the roadside unit for communication improvement.


$$Y_{i}=\left(i_{v}+s_{y}\right)*h_{d}\left(A_{d}\right)+o^{\prime }-\left(c_{x}+a_{c}\right)$$ (10)

**Fig 6:**
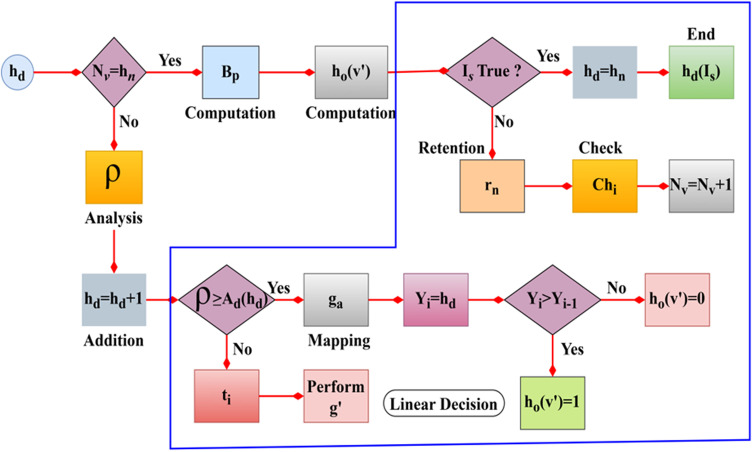
Linear exponent decision for $h_{d}$ **augmentation and availability detection.**

The identification is $Y_{i}$ which is derived from the factor of $c_{x}$ and $L_{g}$ for one instance and they are symbolized as $i_{v}\text{and}s_{y}$. This is used to identify whether the interaction is executed correctly or not. The linear exponent decision for $h_{d}$ augmentation and availability detection is represented in Fig 6.

In Fig 6, the linear decision process is illustrated under $h_{o}\left(v^{\prime }\right),\rho ,$ and $Y_{i}$ conditions. Based on the available $N_{v}$, the $B_{p}$ and $h_{o}\left(v^{\prime }\right)$ computations are linear. In this process, $h_{d}\left(I_{s}\right)$ is terminated only if $c_{x}$ and $A_{x}$ are similar to support $L_{g}$. Depending on the $h_{d}$ augmentation (addition), the vehicle availability is maximized for o' and $ch_{i}$ addressed $L_{g}$. If these conditions are satisfied, then availability is high, for which the linear exponent generates $A_{x}$ flexible $E_{d}$ and $r_{n}$. Therefore, both factors achieve Ve' for maximum $B_{p}$ computed for $h_{n}\in N_{v}$ linearly.

**Fig 7:**
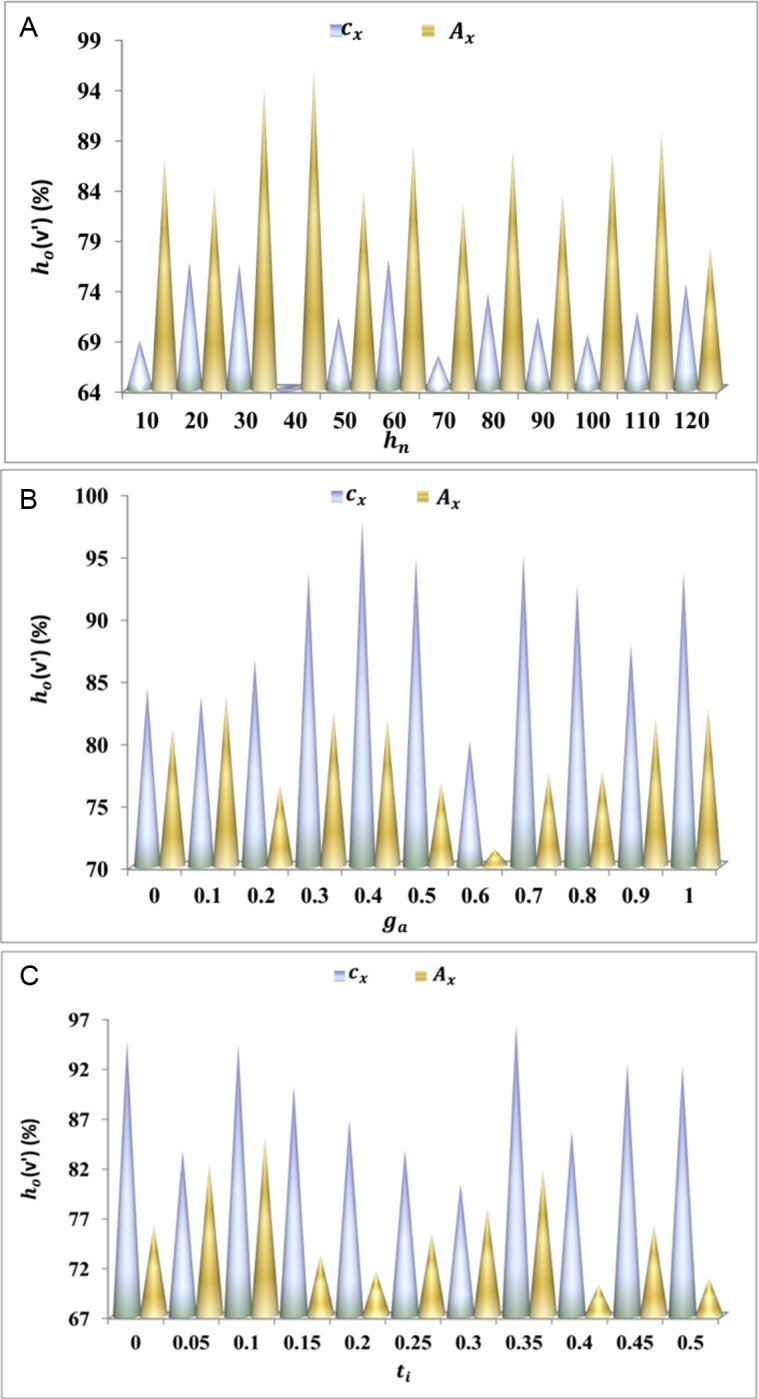
(A–C) $h_{o}\left(v^{\prime }\right)$ **Valuation for different variants.**


The vehicle availability is high, and herd optimization is executed by validating the path changes. From this process, traffic is identified and detects the availability of the vehicle and executes the routing with herd optimization $h_{d}\left(z^{\prime }*N_{v}\right)+v'$. From this step, it indicates the mapping with the preceding data storage in ITS and forwards the transportation of data to the neighbor vehicle, where it is checked for the availability rate $v^{\prime }\left(u_{r}*t_{i}\right)+o'$. This execution step indicates the convergence rate and improves vehicle availability (Fig 7a–7c). Based on this state, the roadside vehicle communicates with the neighbouring vehicle, ensuring longevity and communication. Next comes the validation of higher longevity, which is derived below.

$$V_{l}=\left(L_{g}+a_{c}\right)*\prod_{c_{x}}w_{y}-A_{d}\left(h_{d}\right)+I_{s}$$ (11)

If there is a pathway change, the herd optimization invokes and performs the adding of the herd for the computation, validating is $V_{l}$. This search is carried out for the communication and interaction improvement in smart cities for nnumber of vehicles. This strategy evaluates the interruption and convergence detection and checks for availability.


$$g_{a}=E_{d}-h_{d}\left(T^{\prime }\right)+t_{i}+z'\left(L_{g}\right)$$ (12)

The mapping is followed up with the preceding ITS data storage and produces the resultant. This increases the optimization of herds if pathway changes happen among the vehicles. This factor is designed for higher longevity and communication from the search process. Since it is an integration of herd optimization and linear exponent it is executed in a hybrid manner. This case of evaluation is mapped with the preceding data set and generates the resultant whether the herd is newly produced or not that is used to ensure mobility and longevity. From this observation step, the detection is done to address the convergences failing.


$$\pi \left(f_{i}\right)=\left(Ve^{\prime }-G_{m}\right)+\sum_{N_{v}}\left(a_{c}+z'\right)*g_{a}\left(E_{d}\right)$$ (13)

The detection is done for the failure addressing, and it is described as $f_{i}$, these convergences and interruptions are analyzed for the routing of vehicles and reduce the time lag. Based on detection of failure the convergences are processed and improve the longevity and interaction. By linearly matching the vehicle’s available time based on mobility and longevity, the consecutive herd agents are initiated for new neighbor searches. Therefore, the failing convergence activates a new search process for interaction longevity to confine the paused handoff problem.

**Fig 8:**
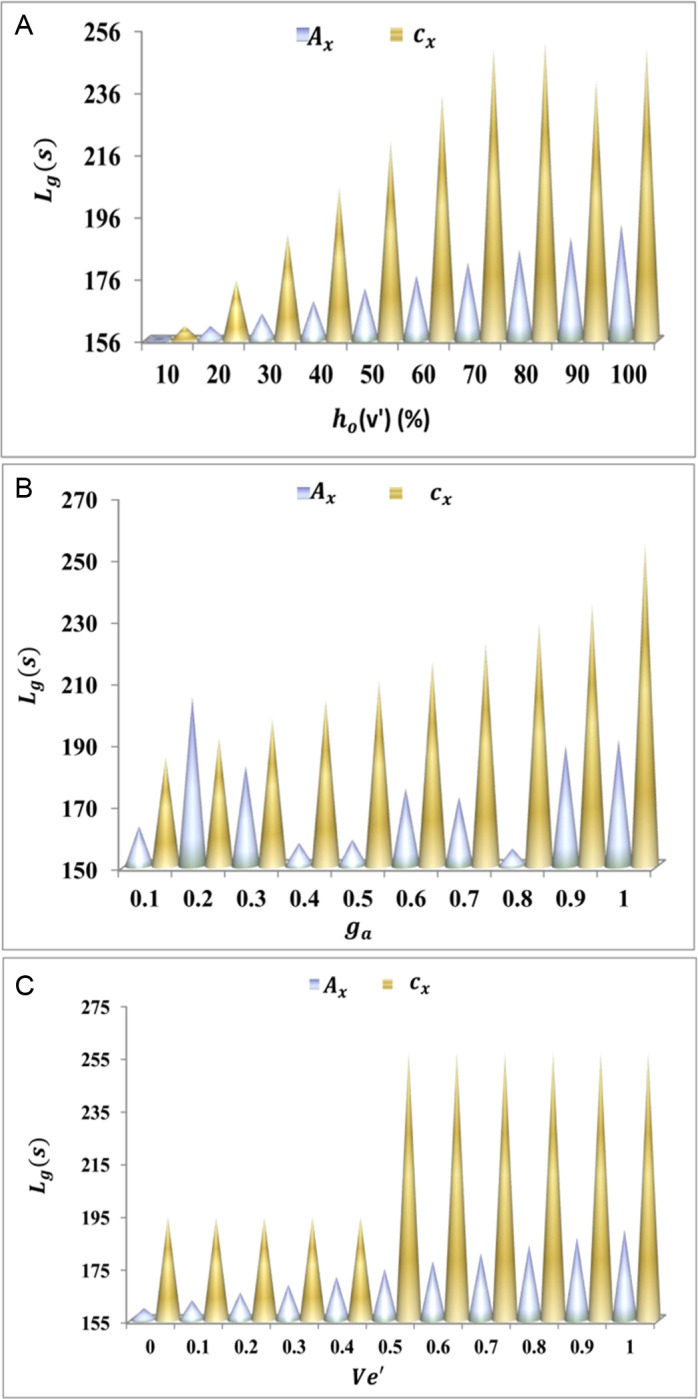
(A–C) $L_{g}$ discussion for $c_{x}$ and $A_{c}$.

The algorithmic process for herd optimization is discussed below. This is required to analyze the$L_{g}$(Fig 8a–8c) for the different variants such that$PH_{o}$are reduced.

**Table:** 

Algorithm 1 Herd Optimization for $L_{g}$
Initialization: $B_{p}+I_{s}\left(v^{\prime }\right)$ for $\left(f_{i}+h_{d}\right)*z'$ do $h_{d}\leftarrow z'\left(t_{r}*Y_{i}\right)$ for periodic checking Calculate $\partial *\left(L_{g}+a_{c}\right)$ for each detection is done in Eq. 3. Calculate $\pi +\left[\left(o^{\prime }+v'\right)*PH_{0}\right]$ for each analysis is processed in Eq. 2. Analysis of $Y_{i}+\left(k_{h}*ch_{i}\right)-G_{m}$ for each convergence is done in Eq. 11. Calculate vehicle $h_{n}\to \left[\left(k_{h}*ch_{i}\right)-G_{m}\right]$ if $V\left[e^{\prime }+h_{n}*\left(T^{\prime }-g_{a}\right)\right]$ then Mapping is carried out $g_{a}\left(E_{d}-T^{\prime }\right)$ in Eq. 12 End if If $g_{a}*\left(v^{\prime }\left(h_{0}+w_{y}\right)\right)$ Else End if End for Return $E_{d}-g_{a}\left(T^{\prime }-Ve^{\prime }\right)$ Update the training given to herd agents.

**Table:** 

Algorithm 2 Availability Estimation
Initialization: $Y_{i}+\left(\partial *f_{i}\right)-G_{m}$ for $\pi +\left(o^{\prime }+T^{\prime }\right)*L_{g}$ > 0 do Examine $w_{y}+\left(Ve^{\prime }-G_{m}\right)$ Measure $\left(E_{d}-N_{v}\right)*g_{a}$ for each $L_{g}-G_{m}$ using Eq. 2. Measure longevity and communication equated in Eq. 10. Calculate the fitness condition in Eq. 5. Measure $T^{\prime }-Ve^{\prime }*\left(z^{\prime }+h_{d}\right)$ for each $c_{x}*g'$ via Eq. 4a. Measure $h_{d}\left(z^{\prime }*\partial \right)+N_{v}-f_{i}$ Detect the convergences $\pi \left(Ve^{\prime }+a_{c}\right)-G_{m}$ Execute the path-changing $\partial \left(w_{y}+E_{d}\right)-PH_{0}$ If $\left(h_{d}+A_{d}\right)*Y_{i}+o'$ then Else if if $A_{d}\left(z^{\prime }-w_{y}\right)-Ve^{\prime }<L_{g}$ end if $PH_{0}-w_{y}\left(h_{n}\right)>E_{d}$ Else if return $B_{p}+(N_{v}*u_{r})$

**Fig 9:**
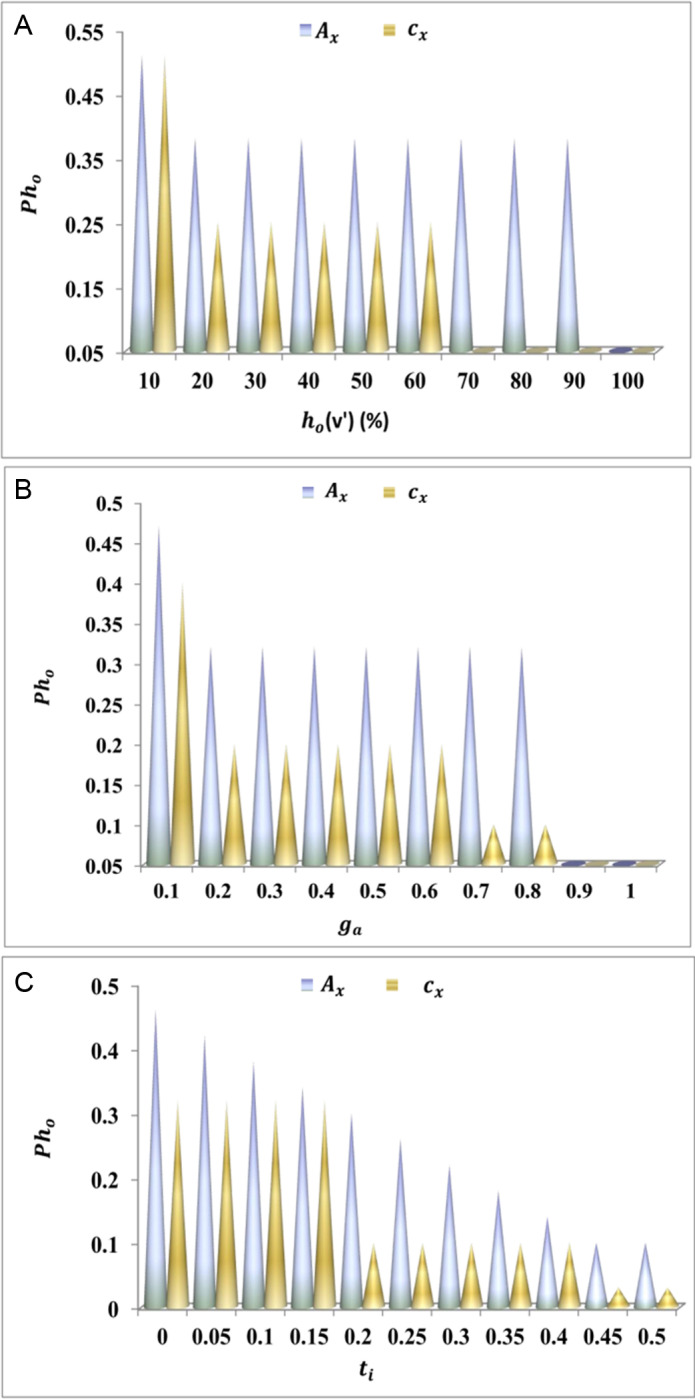
(A–C) $PH_{o}$ assessment for $c_{x}$ and $A_{c}$.

The longevity of the proposed work increases for the average interaction time. This executes the communication and interaction in the higher range where the data transportation is carried forward $o'\left(T^{\prime }+c_{x}+a_{c}\right)$. This examination step indicates the search for a vehicle to transmit the data and optimize the resultant $\partial +\left(h_{0}+o^{\prime }\right)-G_{m}$. This is followed up to decrease the time lag that decreases longevity. So, in this case, it illustrates lesser convergences, and mapping is done with the preceding data in ITS and gives the result as higher longevity (Fig 8a–8c). Following this discussion, the availability estimation is described in Algorithm 2 to reduce $PH_{o}$ as analyzed using Fig 9a–9c.

**Fig 10:**
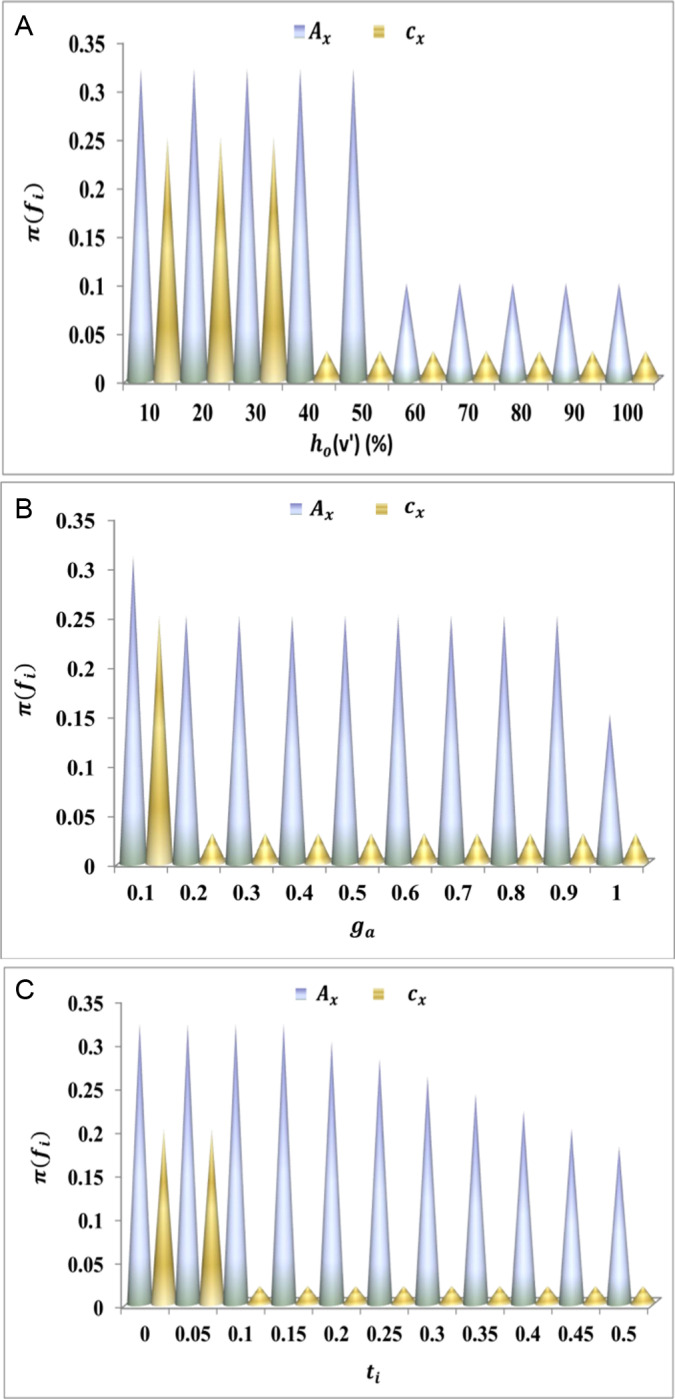
(A–C) convergence failure assessment for $c_{x}$ **and** $A_{c}$.

Paused handoff decreases if the detection of convergences and interruptions is done for the vehicles. On this basis, the path changing occurs due to the herd optimization strategy and ensures transportation without any time lag $o^{\prime }\left(G_{m}-h_{n}\right)*Ve'$. Based on this evaluation step, the interaction and communication lead to the routing of vehicles and decrease the traffic in smart cities $u_{r}\left(h_{0}+c_{x}+a_{c}\right)*k_{h}$. The fitness function is extracted from the hybrid trained herd optimization method and reduces the paused handoff (Fig 9a–9c). The convergence description is presented in Algorithm 3 followed by the convergence failure assessment in Fig 10a–10c.

**Table:** 

Algorithm 3 Convergence Estimation
Initialization: $L_{g}+\left(a_{c}+c_{x}\right)-Ve'$ for $\left(c_{x}+a_{c}\right)*L_{g}$ do Detect $h_{n}+\left(\partial *t_{r}\right)+\pi$ Analysis $o^{\prime }+\left(T^{\prime }-E_{d}\right)*g_{a}-f_{i}$ for each $r_{n}\left(L_{g}\right)-Ve'$ via Eq. 9 Addition of herd optimization $A_{d}+\left(h_{d}*h_{0}\right)+\partial$ for each $N_{v}\left(I_{s}\right)$ using Eq. 7. Detect $PH_{0}+\left(r_{n}-G_{m}\right)$ where $v^{\prime }\left(h_{0}\right)+Y_{i}$ The path changing is carried out $w_{y}\left(h_{0}+f_{i}\right)-N_{v}$ for each via Eq. 6 If $h_{d}+\left(w_{y}-g^{\prime }\right)*\rho$ then Else if If $G_{m}+\left(ch_{i}*PH_{0}\right)>A_{c}$ Else if if $L_{g}-Ve'\left(G_{m}+v^{\prime }\right)$ end if end for Return $A_{c}\left(h_{0}+o^{\prime }\right)>L_{g}$ Update $h_{d}\left(z^{\prime }+Ve^{\prime }\right)*g_{a}\left(N_{v}\right)$

The convergence failure is reduced if the addition of new herd optimization invokes this methodology. In this phase, the analysis is done in the linear exponent manner and a periodic check is carried out for the interaction rate $a_{c}+\left(Ve^{\prime }*f_{i}\right)$. This process executes the new search process for convergence failure and improves the interaction and communication $Ve^{\prime }+\left(a_{c}+c_{x}\right)$. On this basis, the path changing is carried out with the linear exponent function and exhibits the detection without time lag (Fig 10a–10c).

## 4. Results and discussion

The proposed model is experimented with using a VanSim simulation scenario using a random open street map. This map represents a road segment 4 Km long and 7 interactions adaptable to 120 vehicles/ hours. The average vehicle speed is 30 km/hr to 80 Km/hr and the simulation environment is deployed with 5 roadside units covering 500 m. A single vehicle is capable of covering a 300m range with its $N_{v}$. The maximum $N_{v}$ (both static & mobile) observed is 1 and the average $c_{x}$ time is between 10s and 240s for sharing location, navigation, message, etc., information. The $G_{m}$ acceptable is 120 ms after which $PH_{o}$ is experienced; if this $G_{m}>240s$, then the need for $N_{v}$ is high. Similarity, the $H_{d}$ is modelled to be the same as the $N_{v}$ available within the communication range. The no. of comparison methods from existing related works have been considered for analysis like PDMAC [32], CDF-ITS [26], and CDN-ITS [33] in comparison with the proposed CLoIM has been updated in Tables 1 and 2.

**Table 1:** Comparison values of Avg. interaction time.

Vehicles	$G_{m}$ (ms)				$L_{g}$ (s)				Interaction Time (s)			
	PDMAC [32]	CDF-ITS [26]	CNN-ITS [33]	CLoIM	PDMAC [32]	CDF-ITS [26]	CNN-ITS [33]	CLoIM (high)	PDMAC [32]	CDF-ITS [26]	CNN-ITS [33]	CLoIM
20	114.2	112.6	102.6	104.23	161.25	171.25	181.25	187.36	156.3	166.3	169.3	174.36
40	111.1	110.98	108.98	99.58	168.36	178.36	188.36	192.36	165.25	175.25	179.25	185.6
60	102.3	102.3	112.3	87.26	172.36	182.36	192.36	204.36	169.69	179.69	189.69	198.7
80	85.24	76.8	96.8	69.58	178.14	188.14	208.14	212.25	174.36	188.36	198.36	214.36
100	65.74	77.8	97.8	50.3	182.57	192.57	222.57	256.31	181.36	191.36	221.36	225.41

**Table 2:** Comparison values of Avg. handoff outage.

Velocity (Km/Hr)	$B_{p}$				$Y_{i}$ (%)				$PH_{o}$ (s)			
	CDF-ITS [26]	PDMAC [32]	CNN-ITS [33]	CLoIM	CDF-ITS [26]	PDMAC [32]	CNN-ITS [33]	CLoIM	CDF-ITS [26]	PDMAC [32]	CNN-ITS [33]	CLoIM
30	0.814	0.874	0.894	0.904	54.6	61.6	54.6	62.37	17.26	19.26	21.26	12.45
40	0.741	0.841	0.871	0.904	58.32	62.32	56.32	65.81	23.89	21.89	26.89	15.85
50	0.714	0.871`	0.884	0.897	64.87	74.87	67.87	78.96	34.36	29.36	38.36	26.97
60	0.697	0.797	0.827	0.869	69.21	79.21	78.21	82.31	42.36	46.36	43.36	36.71
70	0.654	0.754	0.74	0.785	71.21	81.21	78.21	85.97	44.58	48.58	41.58	38.69
80	0.625			0.715	74.69			88.69	93.04			72.36

The average interaction time increases between the vehicles and decreases the paused handoff mechanism. This approach is used to state the path changing for the vehicle and estimates the higher convergences rate $Ve^{\prime }\left(w_{y}+o^{\prime }\right)*T'$. Executing this step illustrates the higher longevity and provides the periodic checking of neighboring vehicles and shares the information $N_{v}\left(L_{g}+c_{x}\right)*a_{c}$. Based on this part optimization is carried forward and the cooperative mechanism runs on adding herd into the computation and increases the interaction time (Table 1). The average handoff outage time for the proposed model is compared with the existing method and tabulated below.

The average handoff outage decreases for vehicle transportation with higher longevity and communication. This process is used to define the matching with the preceding ITS and produces the output as average handoff which has been reduced. On this evaluation mechanism, the pathway changing occurs if the vehicle transportation fails to add the herd agent into the processing $A_{d}\left(h_{d}+L_{g}\right)*ch_{i}-PH_{0}$. This validation shows lesser average handoff outage for the vehicle routing $u_{r}\left(h_{0}*\pi \right)+\partial$ (Table 2).

## 5. Conclusion

Paused handoff in ITS scenarios is common due to hefty vehicle densities and seamless interaction demands. To address this problem, the cooperative longevity of the interaction model is proposed and discussed in this article. The proposed model utilized herd optimization with linear exponential function for neighbor selection, interaction establishment, and longevity improvement. The cooperative interaction intervals between the neighbors are used to validate the herds based on vehicle availability. Therefore, the herd initialization at the initial stage is random until maximum fitness is identified for selection. Considering this fitness, the convergence is estimated for longevity improvement toward new neighbor discovery. The linear exponential process decides the need for a new herd agent or termination based on mobility, adaptability, and longevity. Therefore, the convergence is validated for its failure from which a new search is pursued. Therefore the conjoined searching and fitness estimation processes are used to reduce the paused handoff problem. This proposed model improved the interaction longevity by 10.81% and reduced the paused handoff lag by 9.17% for the different vehicle densities and velocities.

## 6. Future works

This proposed model experiences the following limitation: waiting for interaction re-establishment post the paused handoff time. This is observed due to the multiple connectivity choices and vehicle unavailability. The integration of communication technologies resolves safety and security issues. Developing strong security mechanisms to safeguard ITS communication and prevent cyber threats should be the primary goal of future research. Additionally, integrating CLoIM with emerging technologies such as 5G and future 6G networks could significantly improve communication capabilities. It is vital to investigate how these advancements can support real-time data exchange. Longitudinal studies evaluating the long-term impacts of CLoIM on traffic flow and safety will provide valuable insights into its sustainability in real-world applications.

Furthermore, incorporating advanced machine learning techniques, particularly reinforcement learning, may optimize communication strategies by predicting traffic patterns and adapting parameters based on real-time conditions. A forehand knowledge-based handoff is to be augmented to the linear exponential function to surpass this limitation. Therefore, it classifies the unavailability and availability constraints to improve the convergence rate further.
